# A Resource-Efficient Hybrid CNN-LSTM Network for Image-Based Bean Leaf Disease Classification

**DOI:** 10.3390/jimaging12100468

**Published:** 2026-09-24

**Authors:** Hye Jin Rhee, Joseph Damilola Akinyemi

**Affiliations:** Department of Computer Science, University of York, York YO10 5GH, UK; hrhee.dev@gmail.com

**Keywords:** bean leaf disease, convolutional neural networks (CNNs), deep learning, EfficientNet, image augmentation, long short-term memory (LSTM)

## Abstract

Accurate and resource-efficient automated diagnosis is a cornerstone of modern agricultural expert systems. While Convolutional Neural Networks (CNNs) have established benchmarks in plant pathology, their ability to capture long-range spatial dependencies is often limited by standard pooling layers, and their high memory footprint hinders deployment on portable devices. This paper proposes a lightweight hybrid CNN-LSTM system for bean leaf disease classification. By integrating an LSTM layer to model the spatial–sequential relationships within feature maps, our hybrid architecture achieves a 94.36% accuracy and 94.38% F1 score while maintaining an exceptionally small footprint of 1.86 MB, a 70% reduction in size compared to traditional CNN-based systems. Furthermore, we provide a systematic evaluation of image augmentation strategies, demonstrating that tailored transformations are superior to generic combinations for maintaining the integrity of diagnostic patterns. Results on the *ibean* dataset confirm that the proposed system achieves state-of-the-art F1 scores of 99.22% with EfficientNet-B7+LSTM, providing a potentially robust and scalable framework for real-time agricultural decision support in resource-constrained environments. The code and augmented datasets used in this study are publicly available on GitHub.

## 1. Introduction

Automated plant disease identification is a crucial smart farming system that helps identify real-time crop infections. In common bean (*Phaseolus vulgaris* L.) plants, prevalent fungal diseases, such as bean rust and angular leaf spot, compromise the plants’ photosynthetic capacity, which can cause structural damage to the plant and extensive loss of yield. For instance, the production loss in Uganda in 2012 caused by bean rust and angular leaf spot was estimated at up to 47% and 55%, respectively [1]. Visual detection remains the essential first step in identifying symptoms, and subsequent microscopic or biochemical component analysis confirms the pathogens. This approach can be expensive and requires processing time [2]. Automated disease identification has increasingly been considered an attractive means to achieve timely agricultural disease management [3].

The practical management of common bean crops requires diagnostic tools that are not only accurate but also economically viable for smallholder farmers. While traditional biochemical analysis is a definitive ’gold standard,’ the associated costs and logistical delays often render it inaccessible for real-time field management [2]. By developing ultra-lightweight architectures that maintain high diagnostic precision while minimising computational overhead, this research provides a potential for Edge-AI deployment. Such systems enable immediate, on-site decision-making, allowing farmers to initiate localised treatments before a minor outbreak scales into the severe yield losses observed in regions like Uganda [1]. This shifts the paradigm from reactive agricultural management to a proactive, data-driven approach that is sustainable even in resource-constrained environments.

The development of automated expert systems for agriculture is increasingly essential as global food security faces threats from climate-driven disease outbreaks. Conventional diagnostic processes rely on the availability of human experts, which is often a bottleneck in large-scale farming or remote regions. An effective agricultural expert system must bridge the gap between high-level diagnostic accuracy and operational feasibility on edge devices. This requires a knowledge-based approach to architecture design, where models are not only deep but also resource-efficient. By integrating sequential logic into spatial feature extraction, an expert system can better mimic the human expert’s ability to contextualise localised symptoms within the broader structural geometry of a leaf, thereby providing more reliable decision support for crop management.

Artificial intelligence (AI) techniques, such as machine learning (ML) and deep learning (DL), have been instrumental in automated bean leaf disease detection. Traditional ML algorithms, such as support vector machines (SVMs) [4], have been used to classify bean leaf disease. DL methods, such as CNNs, have been even more successful in accurately detecting and classifying diseases [5]. Nonetheless, exploring alternative tools beyond conventional CNNs is in high demand. Leveraging CNN with LSTM has shown encouraging results in various computer vision tasks, such as medical imaging [6] and human motion recognition [7]. However, this design has not been extensively explored for common bean leaf disease. Furthermore, previous studies that commonly addressed data shortages by implementing data augmentation [8,9] have yet to systematically review the impact of various schemes. Notwithstanding, the suitability of a technique is highly subject to various aspects of ML operations.

Our primary experiments involved additional data generation using augmentation techniques and constructing two DL architectures: Bean-CNN (baseline CNN) and Bean-CNN-LSTM (hybrid CNN-LSTM). We trained these models on original and augmented images, and then compared their performance across different augmentation strategies. In additional experiments, we further expanded the data and trained fine-tuned EfficientNet [10] models using this hybrid design concept.

The rest of the paper is organised as follows. Section 2 reviews related work. Section 3 describes the methodology employed in our experiments. Section 4 and Section 5 present the experimental results and discussion. The final section summarises our findings.

## 2. Literature Review

### 2.1. Image-Based Automated Plant Disease Identification

With the growing demand for smart farming systems, researchers have proposed techniques for effective image-based plant disease identification. Early research predominantly used traditional ML algorithms combined with feature engineering, as an SVM-based cucumber leaf disease classifier showed the best result with the radial basis function (RBF) [11]. Like many other image classification tasks, adopting CNNs for this problem has significantly improved prediction quality. For example, Lu et al. [12] showed that their compact CNN model outperformed an SVM-based model in their rice disease classification. Naturally, applying DL methods has become a mainstream approach, as Geetharamani and Pandian [13] proposed a nine-layered CNN trained on the Plant Village dataset [14]. More recently, Patil and Manohar proposed an LSTM-CNN tomato leaf disease classifier [15]. A similar attempt by Devi et al. [16] showed superior results for their CNN-LSTM plant disease classifier to existing models. Haque et al. [17] proposed a Vision transformer (ViT) with triplet multi-head attention to tackle disease detection and achieved a 97.99% accuracy on plants and apples.

Due to data scarcity, the common bean plant is less studied among agricultural crops [18]. In most bean disease research, the transfer learning approach using pre-trained models, such as MobileNet [9] or EfficientNet [19], is common. A more recent attempt using YOLO (You Only Look Once) [20] was successful in detecting the damage by bean leaf beetles. The customisation of new deep learning architectures is starting to gain momentum, as an article [8] proposed a combined model using the histogram of oriented gradients (HOGs) and CNN for bean leaf disease identification.

The main drawback of previous attempts is that these complex models have limited usability in real-world situations under hardware constraints [21]. For real-world deployment, we explored the potential of a hybrid CNN-LSTM as a more efficient deep learning architecture than existing models, and this is rarely attempted for the classification of common bean disease.

### 2.2. Image Augmentation in Plant Disease Identification

Data augmentation is commonly applied to image classification problems to improve diversity in training data. The classic model-free approach includes geometric and photometric transformations. Geometric transformations, including rotation, cropping, and flipping, manipulate the geometric primitives of an image. On the other hand, photometric transformations adjust the colour space information, such as RGB (Red–Green–Blue) and HSB (Hue–Saturation–Brightness). The most popular method in plant disease research is the multi-technique combination due to practicality and general consensus over its effectiveness. This strategy involves applying several transformations to the image data proportionally or randomly. The application of this technique in single-crop [22], and multi-crop classifiers [23] demonstrated its effectiveness in generating large training datasets. Similarly to other plant disease studies, this combination technique is a popular approach in bean disease research, typically without reviewing its efficacy. However, various articles [24,25,26] have suggested that the effectiveness of any augmentation can be highly dependent upon the context, such as the type of task or the model. Therefore, we attempt to review several techniques for our custom model development.

## 3. Methodology

In our initial experiments, we constructed lightweight, custom-designed deep learning architectures built from scratch. For these experiments, we expanded the training samples to three times the original training data by implementing data augmentation techniques. We trained and evaluated our models to contrast their performance to determine which architecture and augmentation techniques were superior to others.

Our experiments were carried out on a hardware environment with an Intel Xeon CPU running at 2.2 GHz and an NVIDIA Tesla 4 GPU. We used OpenCV (v 4.10) and Scikit-image (v 0.23.2) packages for augmentation and TensorFlow (v 2.17) for model construction.

### 3.1. Data Preparation and Augmentation for Custom Lightweight Models

The *ibean* data [27] is created by Makerere AI Research Lab and contains field-collected bean leaf images showing two disease classes and one healthy class, as shown in Figure 1. Whilst angular leaf spot displays distinctive, angular-shaped damage [28], bean rust exhibits irregular light haloes, which may progress to darker blotchy patterns.

The originally downloaded (OD) *ibean* dataset contained 1295 images (excluding one corrupted image file), divided into 1034 training images, 133 validation images, and 128 test images. We first combined all splits in the OD dataset into one, shuffled it, and then split it into 70% (905 samples) for training, 15% (195 samples) for validation, and 15% (195 samples) for testing. The new 70% training set is henceforth referred to as the unaugmented training set. Only the unaugmented training set (70%) was augmented; the validation and test subsets did not contain augmented data. This combination and re-splitting of the dataset was carried out to create larger and identical validation and test sets, as seen in Table 1. To obtain the augmented training sets, we applied 5 different augmentation operations to the unaugmented training set. Each augmentation operation was applied to each of the three dataset classes (Angular leaf spot, Bean Rust, and Healthy) using two different parameters to increase variability. Thus, to obtain the five augmented training sets, we applied augmentation operations as follows:

1. Brightness: Increase the brightness on a linear scale by factors of 20 and 30. Each scale factor is applied to ≈50% of each dataset class.
2. Crop: By ratios of 0.8 and 0.9. Each crop ratio is applied to ≈50% of each dataset class.
3. Flip: Horizontally and vertically. Each flip direction is applied to ≈50% of each dataset class.
4. Rotation: 15° clockwise and 15° counterclockwise. Each rotation angle is applied to ≈50% of each dataset class.
5. Combination: Random combinations of all 4 of the operations above.

**Table 1:** Dataset distribution: originally downloaded (OD), unaugmented and augmented.

	Classes			
Data Partition	Angular Leaf Spot	Bean Rust	Healthy	Total
Originally downloaded (OD)	432 (33.36%)	436 (33.67%)	427 (32.97%)	1295
Train (OD)	345 (33.37%)	348 (33.66%)	341 (32.98%)	1034
Val (OD)	44 (33.08%)	45 (33.83%)	44 (33.08%)	133
Test (OD)	43 (33.59%)	43 (33.59%)	42 (32.81%)	128
Unaugmented Train	304 (33.59%)	307 (33.92%)	294 (32.49%)	905
Unaugmented Val	60 (30.77%)	67 (34.36%)	68 (34.87%)	195
Unaugmented Test	68 (34.87%)	62 (31.79%)	65 (33.33%)	195
Brightness	912 (33.59%)	921 (33.92%)	882 (32.49%)	2715
Crop	912 (33.59%)	921 (33.92%)	882 (32.49%)	2715
Flip	912 (33.59%)	921 (33.92%)	882 (32.49%)	2715
Rotation	912 (33.59%)	921 (33.92%)	882 (32.49%)	2715
Combination	912 (33.59%)	921 (33.92%)	882 (32.49%)	2715

This resulted in 2715 samples for each augmented training set, all with identical class distributions to that of the training set, as shown in Table 1. In Table 1, the train (OD), val (OD), and test (OD) refer to the data partitions available in the OD dataset, while unaugmented train, unaugmented val, and unaugmented test refer to the unaugmented partitions created from re-splitting. All data splitting, shuffling, and augmentation at this stage were controlled by a random seed of 0. Samples of the effect of each augmentation technique on one image is shown in Figure 2

### 3.2. Custom Deep Learning Architecture

The idea of integrating a CNN and a recurrent neural network (RNN) was first introduced by Deng and Platt [29] for speech recognition. This approach has been considered efficient [30] and has been shown to be effective for human-activity recognition [31].

The theoretical justification for incorporating an LSTM layer into a computer vision pipeline lies in treating spatial feature maps as a pseudo-temporal sequence, an established paradigm in spatial sequence modelling [32,33]. This is even more recently demonstrated in successful vision literature such as the Vision Transformer [34]. While standard CNNs excel at extracting local hierarchical features through localised convolutional kernels, they often lack an explicit mechanism to capture long-range relational dependencies across the spatial grid without adding deep stacks of pooling or parameter-heavy dense layers. By unrolling the spatial feature grid into a sequence of feature vectors, the LSTM cell acts as a contextual accumulator over adjacent spatial regions. Its gating mechanisms systematically preserve spatial continuity across contiguous rows while modulating transitions across boundaries, allowing the network to model directional Markov dependencies across the leaf geometry. In bean leaf pathology, where lesions like angular leaf spot manifest in sporadic but structurally correlated patterns, this spatial-to-sequential integration contextualises localised symptoms within broader leaf spatial patterns, providing a richer structural representation than isolated fully connected projection layers.

Figure 3 presents our proposed model architecture. This is a VGG-inspired [35] DL network that offers greater flexibility in modifying the fully connected (FC) layers. We selected this stacked architecture because it has the advantage of capturing intricate patterns in an image over residual networks [36]. Small filters are selected for computational efficiency, and padding parameters are used to minimise the boundary effects. We implemented either the stride parameter or max-pooling to reduce the input resolution and improve feature extraction [37]. All convolutional layers employ ReLU combined with the Kaiming (He) initialisation to increase non-linearity and ensure more reliable convergence [38].

To effectively leverage the LSTM’s capability for spatial correlation, we transform the 2D feature maps generated by the final convolutional layer into a structured 1D temporal sequence. Instead of a standard flattening operation, which collapses the spatial hierarchy, the feature maps are reshaped into consecutive time steps that represent a scan-line or patch-wise traversal of the leaf image. This allows the LSTM’s internal gates, specifically the forget ($f_{t}$) and input ($i_{t}$) gates, to learn the contextual dependency between adjacent pixel regions. In agriculture, where disease manifestations such as bean rust spread in sporadic and blotchy clusters, the LSTM acts as a secondary filter that identifies the recurring geometric patterns of pathogens across the entire leaf surface, leading to a potentially improved performance gain over baseline CNNs. Hence, whilst the vectors fed into the densely connected neural network (NN) in Bean-CNN were flattened to preserve spatial information, these vectors were sequentially reshaped in time steps for Bean-CNN-LSTM. An LSTM is a modified variant of RNNs [39] and uses the backpropagation through time (BPTT) technique in its cell-based computations, allowing the gradient update across multiple time steps. The relevant LSTM computations are expressed in the following equations:

(1) $$f_{t}=\sigma \left(W_{f}x_{t}+U_{f}h_{t-1}+b_{f}\right)$$

(2) $$i_{t}=\sigma \left(W_{i}x_{t}+U_{i}h_{t-1}+b_{i}\right)$$

(3) $${\tilde{c}}_{t}=\tanh\left(W_{c}x_{t}+U_{c}h_{t-1}+b_{c}\right)$$

(4) $$c_{t}=f_{t}⊙c_{t-1}+i_{t}⊙{\tilde{c}}_{t}$$

(5) $$o_{t}=\sigma \left(W_{o}x_{t}+U_{o}h_{t-1}+b_{o}\right)$$

(6) $$h_{t}=o_{t}⊙\tanh\left(c_{t}\right)$$

where $f_{t},i_{t},o_{t}\in R^{H}$ represent the forget gate [40], input gate, and output gate activation vectors at time step *t*. ${\tilde{c}}_{t}\in R^{H}$ is the candidate cell state, and $c_{t}\in R^{H}$ is the updated cell memory state vector. $h_{t}\in R^{H}$ is the hidden state output vector passed to subsequent layers/steps. $W_{*}\in R^{H\times D}$ and $U_{*}\in R^{H\times H}$ denote the input-to-hidden and recurrent hidden-to-hidden weight matrices, respectively. $b_{*}\in R^{H}$ represents the bias vectors. σ(·) is the element-wise logistic sigmoid function, tanh(·) is the hyperbolic tangent activation, and ⊙ denotes the Hadamard (element-wise) product.

To examine the quality of the estimated weights, our proposed models use the softmax and sparse categorical cross-entropy loss functions. The probability calculated by softmax is integrated with categorical cross-entropy to approximate the prediction error. The following equation describes the computation of this loss function *L*, where $v_{k}$ is the prediction in *k* classes for the $j_{th}$ class of the $i_{th}$ data point and $y_{i}$ is the true distribution.

(7) $$L=-\sum_{i=1}^{k}y_{i}log\left(\frac{e^{v_{i}}}{\sum_{j=1}^{k}e^{v_{j}}}\right)$$

For model evaluation, we collected accuracy, the confusion matrix, the F1 score, and the Matthews correlation coefficient (MCC) for a more balanced analysis [41,42]. The confusion matrix represents the instances of the predicted label against the true label. Correctly predicted instances are true positives (TP) and true negatives (TN). In contrast, false positives (FP) and false negatives (FN) represent erroneous predictions. Based on the confusion matrix, F1 and MCC can be calculated as follows:

(8) $$Precision=\frac{TP}{TP+FP}$$

(9) $$Recall=\frac{TP}{TP+FN}$$

(10) $$F1=2*\frac{Precision*Recall}{Precision+Recall}$$

(11) $$MCC=\frac{TP*TN-FP*FN}{\sqrt{\left(TF+FP)(TP+FN)(TN+FP)(TN+FN\right)}}$$

## 4. Experiments and Results

### 4.1. Experimental Setup for Custom Lightweight Models

To ensure the best performance and a balance between overfitting and underfitting, we optimised each model’s capacity, such as the number of hidden layers and units. Table 2 describes the optimal parameter and hyperparameter values that we found during the iterative fine-tuning process for our custom models. The two values seen for the sequential layer in Table 2 correspond to the number of hidden units for the first and second sequential layers in each custom model.

To evaluate stability and rule out performance artefacts from lucky weight initialisations, training experiments were repeated across 30 independent runs on each of the six training sets (the unaugmented dataset and the five augmented training sets). Each run was governed by a distinct random seed (integers 1 to 30), which controlled both the pseudo-random weight initialisation (using the He initialisation [38]) and mini-batch shuffling order during training. The same fixed sequence of 30 seeds was applied across all experimental conditions (the unaugmented dataset and the five augmented training sets) to ensure rigorous and reproducible comparisons. As only the unaugmented 70% training set was augmented to obtain the five augmented training sets, the test and validation sets remained fixed at 15% each across all runs to maintain consistent evaluation conditions.

We used Gradient-Weighted Class Activation Mapping (Grad-CAM) [43] to visualise the features extracted in the convolutional layers. The weight distribution is represented in a heat map in Figure 4 (in the Viridis scale) and shows that the feature extraction by the custom models becomes more specific in deeper layers.

### 4.2. Results: Bean-CNN vs. Bean-CNN-LSTM

Figure 5 and Figure 6 visualise the training progress of the best models trained on the unaugmented training set (905 samples). Table 3 describes the best performance across all training sets (the unaugmented and the augmented), which is summarised in Figure 7. Bean-CNN-LSTM consistently outperforms Bean-CNN, and the accuracy varies by 2.56% to 5.64%, depending on the training set. A smaller disparity between accuracy and MCC in Bean-CNN-LSTM implies that this model made fewer false predictions than the Bean-CNN models.

We also analysed the results from all 30 training runs and present summary statistics in Table 4, showing the mean and standard deviation values of the accuracy, F1 score, and MCC. Table 4 shows relatively low standard deviations from the mean (in the order of $10^{-2}$) across all three metrics (accuracy, F1, and MCC), indicating a tight distribution. Across all three metrics, the mean values consistently indicate significant improvement of Bean-CNN-LSTM over Bean-CNN. Also, Figure 8 presents the accuracy values in box-and-whisker plots, showing the key statistics of the distribution. The box plots show relatively tight interquartile ranges (IQR) with few outliers. Together, Table 4 and Figure 8 indicate that the distribution is tight and free of skewness and excessive outlier values. From the box plots, it is clear that the IQR of the accuracies obtained by the CNN-LSTM model consistently ranks higher than that of the baseline CNN model, implying that the hybrid CNN-LSTM is more effective than the baseline CNN, with noticeable gaps.

To further evaluate the statistical stability and significance of the CNN-LSTM model over the baseline CNN model, we performed a paired two-tailed *t*-test of the metric values (Accuracy, F1 Score, and MCC) across all 30 experiment runs. The results are presented in Table 5, showing high statistical significance generally in the order of $10^{-2}$–$10^{-7}$. This confirms that the performance gain offered by the CNN-LSTM model is based on statistical significance and mathematical soundness, rather than mere chance or experimental noise.

### 4.3. Results: Effect of Augmentation on Custom Lightweight Models

Training on the augmented sets, as shown in Table 3, generally increases the initial accuracy to a different extent. Bean-CNN shows the highest accuracy on the rotation set, increasing the initial accuracy by 5.13%. The best Bean-CNN-LSTM improves accuracy by 6.67% on the flip set. Both models on the brightness set show the least increase in accuracy with elevated loss. The multi-technique combination also raises accuracy and loss simultaneously, indicating greater uncertainty in their performance [44].

The average performance results of our custom models also show notable trends. As shown in Figure 9, the crop, flip, rotation, and combination methods equally improve Bean-CNN. The loss improves further with the crop technique, indicating an additional benefit to the performance. For Bean-CNN-LSTM, both the flip and the crop techniques are more effective than others when all metrics are considered. Data analysis of the training sets does not show strong correlations between each training set and performance, although we found that the brightness set has elevated frequency and noise levels, which may have negatively contributed to the performance of all models.

In the box plots of Figure 8 and Figure 9, a few training set runs (such as the crop and flip augmented sets) exhibit overlapping or superimposed outlier points beyond the standard 1.5× IQR whiskers. These overlapping data points occur because multiple independent experimental runs (30 seeds) converged to identical discrete test metric values (e.g., exact test accuracies due to the finite test set size of 195 samples, where each single sample misclassification changes accuracy in about 0.51% increments). From an optimisation standpoint, these low-performing outliers reflect instances where specific stochastic weight initialisations coincided with aggressive spatial augmentations (such as severe cropping), leading the network into sub-optimal local minima during gradient descent. The fact that these occurrences are rare (yielding superimposed points outside the upper/lower whiskers while the interquartile boxes remain tightly clustered) underscores the overall stability of the models across the vast majority of initialisation seeds.

From a data governance perspective, these results highlight the necessity of principled data curation over exhaustive data expansion. Our findings indicate that while generic augmentation combinations are often viewed as a default for increasing model robustness, they can introduce a form of synthetic noise that degrades the internal consistency of lightweight models. For agricultural datasets, data governance must prioritise transformations that preserve the biological integrity of the specimen. Geometric shifts like flipping and cropping mimic natural variations in camera angles while maintaining the distinctive morphological features of the lesions. In contrast, excessive photometric noise can obscure the subtle colour gradients critical for distinguishing early-stage rust from nutrient deficiencies. Thus, managing the quality of synthetic data through tailored augmentation is as critical to system reliability as the volume of the underlying training set.

### 4.4. Results: The Best-Performing Custom Lightweight Model

The best-performing custom model was the Bean-CNN-LSTM trained on the flip training set. It showed an accuracy of 94.36%, a weighted average F1 score of 94.38%, and an MCC of 91.64%. Table 6 describes its class-specific performance. The recall score indicates the most false-negative cases in the angular leaf spot class. The precision score shows that the bean rust class has the most false-positive cases. The F1 scores suggest that the most balanced performance is in the healthy class, whereas the least is in the bean rust class. The confusion matrix in Figure 10 visualises this performance and shows a higher error pattern in the bean rust class.

### 4.5. Results: Comparison with Existing Models

To investigate the robustness of our CNN-LSTM-based method, we conducted further experiments with a pre-trained deep network, EfficientNet-B7 [10], trained on a larger version of the *ibean* dataset. We selected the EfficientNet for its excellent performance in image recognition, yet improved efficiency compared to other CNN-based models. The B7 model is even smaller/faster and achieves better image recognition accuracy.

To create an even larger dataset suitable for deep learning, the originally downloaded (OD) *ibean* dataset (see first four rows of Table 1) was augmented to create 40 times larger training and validation sets. To perform this, we used the data splits as found in the OD *ibean* dataset—1034 training images, 133 validation images, and 128 test images. Using the random seed of 0, we augmented only the training and validation sets and retained the 128 test images available in the OD dataset to facilitate fair comparisons with previous works. Thus, no augmented versions of the same image were contained in different splits. We used geometric and photometric transformations, including random rotations, horizontal and vertical flips, scaling, blurring, and contrast/brightness adjustment. Much like the originally downloaded (OD) dataset, the resulting augmented dataset is nearly balanced across the classes, containing a total of 41,360 training imageshl—13,800, 13,920, and 13,640 images for angular leaf spot, bean rust, and healthy leaves, respectively. It also contains 5320 validation imageshl—1760, 1800, and 1760 images for angular leaf spot, bean rust, and healthy leaves, respectively. The augmented dataset and code are available on GitHub (https://github.com/HJin-R/bean_disease)

To allow for some comparison between conventional Dense layers and LSTM layers, we created two separate models from EfficientNet by replacing its final classification layer. In the first model, we replaced the final classification layer with Dense or fully-connected (FC) layers, interspersed with Dropout (30–50%) and batch normalisation layers. In the second model, the classification layer is replaced with an LSTM layer and a linear classification layer, and a Dropout layer (50%) in between them. We refer to the former model as the EfficientNetB7+FC model and the latter as the EfficientNetB7+LSTM model. For best results, the entire architecture was trained, but the EfficientNet backbone was unfrozen in blocks after every five epochs. Both models were trained with the Adam optimiser at a learning rate of 5 $\times \;10^{-4}$, cross-entropy loss function, a batch size of eight and an image size of 380 × 380. The Adam optimiser has proven to be more efficient for image recognition/classification tasks than most other variants of the gradient descent algorithm, such as Stochastic Gradient Descent (SGD) and RMSProp. This is due to Adam’s adaptive learning rate and momentum-based weight updates, which lead to faster convergence [45]. The very low learning rate slowed down the training but allowed for smoother training with more chances of finding the global minima and better generalisation. The batch size of eight was necessary to allow for more memory management and better generalisation while training a very deep network like EfficientNet, but it also slows down training. We chose to increase the image resolution a bit more here to allow for improved performance, i.e., more pixels for convolution and sequencing.

The resulting performances of the models are not unexpected. Table 7 shows the results of both models on the test set; they both reached overall accuracies and F1 scores of 99.22% and had the same scores (precision, recall, and F1) across the classes. Both models reached training and validation accuracies of about 98%, but the EfficientNetB7+FC model reached this performance in only 3 epochs, while the EfficientNetB7+LSTM model reached it in 16 epochs. This means that the FC-based model did not even require the first block of layers of the EfficientNet backbone to be unfrozen before it reached such an excellent classification performance, while the LSTM layer needed some blocks of layers of the backbone to be unfrozen. This shows that the linear layers in the FC-based model are more effective in leveraging the features from the EfficientNet backbone to achieve both high accuracy and efficiency in the bean leaf disease classification task, while the fact that the LSTM-based model achieved the same result with more training indicates that it could not efficiently leverage the huge image features from the EfficientNet backbone for image classification. In fact, to simulate more similarity with the FC-based model, we also included batch normalisation and adaptive average pooling in the LSTM-based model, but the result was rather degraded, reaching only 97.68% test F1 score after 16 epochs. This could also be due to the combined augmentation techniques used in the extended dataset, as our earlier results indicate that the combination of several augmentation techniques did not necessarily produce the best result for both models. However, the fact that the EfficientNetB7+LSTM model still reaches the same accuracy as the EfficientNetB7+FC model after more training epochs indicates that it is suitable for the bean classification task and indeed smaller in size.

Table 8 shows a comparison of the performance of previous models on the *ibean* dataset, with the last three rows referring to our models. To facilitate a fair comparison, we evaluated our models on the standard 128-sample test split provided in the originally downloaded (OD) *ibean* dataset, which matches the test benchmark utilised by most prior works. For each reference model in Table 8, we report the best overall accuracy and overall F1 score exactly as published; where an overall metric was not explicitly reported, it is indicated as “–”. For instance, while Ref. [8] reported individual class-level F1 scores, the authors did not publish an aggregated overall F1 score across all three classes, so no overall F1 score is listed in Table 8. This strict adherence ensures no estimated or inferred values are introduced into the comparative baseline. It is important to note that published methodologies on the *ibean* dataset employ varying data augmentation strategies and training sample sizes to optimise test performance. In benchmark evaluations, data augmentation choices constitute an integral part of each method’s overall training protocol. To ensure rigorous and unbiased evaluation across these diverse pipelines, most of the comparative models in Table 8 are evaluated against the standardised, unaugmented 128-sample test split. Providing the specific training volumes and augmentation types alongside test metrics in Table 8 ensures full transparency regarding the operational conditions under which each reported result was achieved.

It is noteworthy that in many of these works, such as Refs. [8,48,50], the models were trained for a high number of epochs: 100, 50, and 30, respectively. This is most likely because they (except [50]) used the transfer learning approach of training only a few custom layers on top of the pre-trained backbone architecture. While this approach can be computationally less expensive, it does not guarantee the best performance and is prone to overfitting due to the generic image features transferred from the backbone; hence the need for longer training. Standard pre-trained backbones used in prior works range from lightweight edge networks (MobileNetV2: 3.5 M parameters; DenseNet121: 7.9 M parameters) to heavy architectures (InceptionV3: 24 M parameters; EfficientNetB6: 43 M parameters); this places our custom EfficientNetB7 models (65–67 M parameters) in clear context. Overall, both of our pre-trained models (EfficientNetB7+FC, EfficientNetB7+LSTM) achieved better generalisation in fewer training iterations than the previous models.

### 4.6. Ablation Experiments

To evaluate the specific design choices and empirical assumptions of our proposed framework, we conducted three targeted ablation and control experiments on the standardised Seed 0 test split (N=195). These controlled evaluations isolate the contribution of each architectural component, validating the choice of spatial traversal strategies, structural parameter scaling, and data volume constraints. First, we examine the effect of spatial traversal strategies on sequence formation to justify our row-major scanning approach. Second, we evaluate a parameter-matched control model to confirm that performance gains stem from sequential feature integration rather than increased capacity. Finally, we analyse model scaling behaviour across varying training set sizes and augmentation pipelines to demonstrate the operational boundaries of lightweight edge architectures compared to deep pre-trained backbones. All evaluations used the 905/195/195 (Train/Val/Test) partition under Seed 0. As the “Flip” training set consistently gives the best performance for the proposed Bean-CNN-LSTM, all ablation experiments, except the third one, used the “Flip” training set. The third ablation experiment used the unaugmented 905 samples, the 2715 flip set, as well as the augmented 40× larger training set.

#### 4.6.1. Spatial Traversal Strategy Ablation

To evaluate the effect of spatial feature sequence ordering, we tested three traversal methods to flatten the 10×10×64 CNN feature map into time steps. We represent the time steps as *T* and the dimensions as *D*:

1. Row-Major (Scan-Line) [Proposed]: Top-to-bottom row scan (T=100,D=64).
2. Column-Major: Left-to-right column scan (T=100,D=64).
3. Patch-Wise (2×2): Spatial block grouping (T=25,D=256).

As seen in Table 9, continuous line-by-line scanning strategies outperformed coarse spatial block grouping, and row-major order yielded optimal feature alignment compared to the other two strategies.

#### 4.6.2. Parameter-Matched Model Architecture Analysis

To confirm that performance gains stem from recurrent temporal aggregation rather than arbitrary parameter scaling, we designed Bean-CNN-Compact as a control model. This control model replaces the dense projection layers of the standard Bean-CNN (527,171 parameters/6.11 MB) with a compact bottleneck (four dense layers: 5 units → 247 units → 19 units → 3 units) that matches the exact 155,219 parameters (1.86 MB) of Bean- CNN-LSTM.

As shown in Table 10, reducing parameter capacity in a standard feed-forward CNN (Bean-CNN-Compact) causes performance to drop to 82.56% accuracy. In contrast, Bean-CNN-LSTM uses the same 155,219 parameter footprint to achieve 89.74% accuracy. This proves that the recurrent structure enables parameter reduction while simultaneously increasing feature representation power.

#### 4.6.3. Controlled Data Sub-Sampling and Augmentation Analysis

To confirm that performance trends are consistent across dataset scales and are not artefacts of data volume, all four model configurations were evaluated across controlled sub-sampled training partitions: 25%, 50%, and 100% of the 905 unaugmented training images, the 2715 flip set, and the 40× larger augmented set. All models were evaluated on the fixed Seed 0 split (N=195) test set as used in previous ablation experiments.

The training data progression spans five controlled runs as follows:

1. Sub-sampled unaugmented runs (25% and 50% of the 905 unaugmented training images): Evaluates minimal sample availability (226 and 452 unaugmented images).
2. Full unaugmented dataset (100% of the 905 unaugmented samples): Evaluates baseline performance on the raw unaugmented dataset.
3. Small augmented set (2715 “Flip” set): Evaluates the impact of the training data volume on model performance using the same augmented set on which Bean-CNN-LSTM has its best performance.
4. Complete 40× larger augmented dataset: Evaluates the complete pipeline incorporating the same geometric and photometric transformations used in the EfficientNet experiments.

Table 11 reveals the following three major insights:

1. Persistent Architectural Advantage: Bean-CNN-LSTM consistently outperforms Bean-CNN across every run (+2.88% at 25% data, +3.07% at 50% data, +4.02% at 100% data, +5.82% on the 2715 augmented set and +4.56% at the 40× augmented set). This proves that spatial-to-sequential feature integration provides a scale-invariant benefit.
2. Justification for Data Augmentation and Parameter Sizing: On limited training subsets (25% to 100% unaugmented and 1275 augmented data), EfficientNet-B7 performances scale from 78.46% to about 96%. It only reaches its peak performance of 99+% when trained on the full augmented dataset, highlighting the heavy data requirement of its 65 M parameters; hence the need for the 40× larger augmented set. Even with the 1275 augmented images, the EfficientNet models could not achieve such high performance, indicating the need for a larger augmented training set.
3. Justification for a pre-trained model: Despite the 40× larger dataset, the baseline models (Bean-CNN and Bean-CNN-LSTM) reached their peak performances at around 91% and 96%, respectively, while the EfficientNet models reached 99%. The deep backbone of the EfficientNet models gives them a significant edge over the baseline models, especially when the training dataset is large.

**Table 11:** Test Performance across different training and aumentation sizes. All metric values are presented as “Accuracy; F1 score”.

Architecture	Param. Count	25% Train—226 Samples	50% Train—452 Samples	100% Unaugmented Train—905 Samples	2715 Augmented Set (Flip)	40× Augmented Dataset
Bean-CNN	527.2 K	68.91%; 68.85%	76.33%; 75.90%	83.60%; 83.32%	88.62%; 88.48%	91.18%; 90.90%
Bean-CNN-LSTM	155.2 K	71.79%; 71.25%	79.41%; 79.10%	87.62%; 87.36%	94.44%; 94.34%	95.74%; 95.62%
EfficientNet-B7-FC	67.8 M	78.46%; 78.12%	83.59%; 83.40%	90.93%; 90.81%	96.44%; 96.40%	99.36%; 99.28%
EfficientNet-B7-FC	65.2 M	78.46%; 78.10%	83.49%; 83.42%	90.90%; 90.80%	95.95%; 95.71%	99.32%; 99.26%

Together, these three ablation experiments validate the core design principles of the proposed framework. The spatial traversal strategy ablation (Table 9) proves that unrolling feature maps in a scan-line sequence maximises pseudo-temporal coherence for spatial feature extraction. The parameter-matched model architecture check (Table 10) confirms that performance gains stem directly from recurrent spatial feature integration rather than increased parameter capacity, allowing Bean-CNN-LSTM to achieve 89.74% accuracy while matching the ultra-compact 155K-parameter footprint of a bottlenecked CNN (82.56%). Finally, the controlled data scaling experiment (Table 11) highlights the strong relationship between model complexity, feature representation, and dataset scale: while Bean-CNN-LSTM consistently outperforms Bean-CNN across all training subsets (+3.08% to +5.12%), the deep pre-trained EfficientNet-B7 backbones require the full 40× larger augmented dataset to unlock their peak 99+% accuracy. Ultimately, while transfer learning backbones maximise raw accuracy on large datasets, Bean-CNN-LSTM provides an optimal trade-off, delivering high classification fidelity with less than 0.25% of the parameter footprint required by heavy backbones, making it potentially desirable for edge devices.

## 5. Discussion

As summarised in Table 12, adopting an LSTM has a strong advantage for our ultra-lightweight custom models. This hybrid architecture can potentially be an optimal solution in resource-constrained hardware environments such as embedded/edge devices. Note that in Table 12, the EfficientNetB7+LSTM model has two different values in the third column because the number of training parameters increased after more layers of the EfficientNetB7 model were unfrozen during training. However, the remarkably high accuracy despite the smaller model size/number of parameters indicates that the LSTM-based model is an effective approach.

Beyond technical performance, the deployment of this lightweight CNN-LSTM architecture offers potentially significant advantages for integrated agricultural data management. By achieving state-of-the-art results with a model size significantly smaller than traditional CNNs (1.86 MB vs. 6.11 MB for our custom models), this system could potentially facilitate the real-time processing of leaf health data directly on edge devices. In a management context, this decentralised data processing reduces the reliance on costly cloud infrastructure and high-bandwidth connectivity, which are often absent in the rural regions where bean production is most vital. Consequently, this approach could enable more agile farm management decisions, allowing for immediate localised interventions that can mitigate the estimated 47% to 55% yield losses typically associated with bean rust and angular leaf spot. While the compact parameter footprint (1.86MB) of Bean-CNN-LSTM significantly reduces storage requirements and theoretical computational overhead compared to heavy backbones, we note that physical edge deployment involves additional hardware constraints. Factors such as memory bandwidth, operational latency (ms/sample), and energy consumption vary across physical edge accelerators. Thus, our model represents a parameter-efficient candidate design whose real-time performance and power metrics are subject to validation on physical edge hardware in future work.

The dynamic weight computations of an LSTM clearly showed strength in discovering spatiotemporal correlations within the feature map (Figure 11) in the *ibean* data. Our experiment with input vectors resulted in a reduction in the number of features. Despite this, Bean-CNN-LSTM required up to 1.7 times longer training time than Bean-CNN. Nevertheless, Bean-CNN is a more complex model with probably redundant connections [51], which may explain the struggle of this model to achieve the same level of performance as Bean-CNN-LSTM.

To understand why the LSTM layer yields different effects across model families (baseline CNN versus EfficientNet), we must consider the feature extraction capability of the underlying backbone architecture. In lightweight models like Bean-CNN, the feature extractor generates relatively low-level, compact spatial feature maps. Converting these maps into a sequential structure allows the LSTM to act as a temporal feature aggregator and regularizer, helping the model capture contextual dependencies across spatial regions that the baseline CNN missed. This explains the consistent performance improvement of Bean-CNN-LSTM over Bean-CNN. In contrast, a deep, highly expressive backbone like EfficientNet-B7 already extracts rich, highly disentangled high-level representations on its own. As a result, both EfficientNetB7+FC and EfficientNetB7+LSTM reach the same upper limit of 99.22% performance, making the sequential modelling of the LSTM redundant. Furthermore, adding the recurrent parameters of the LSTM head increases optimisation complexity, which is why the simpler Fully Connected (FC) head converges in fewer training epochs. Therefore, while sequential feature modelling offers clear benefits for parameter-constrained custom architectures like the baseline CNN of Bean-CNN-LSTM, a standard FC classification head is more practical and computationally efficient when paired with deep, pre-trained backbones.

Despite the strong performance of our compact models, we observed limitations of these models and the superior and more sophisticated performance of the pre-trained models in this task. More precisely, the best Bean-CNN-LSTM showed the weakest performance in detecting bean rust. Through visual examination of the data, we found that the bean rust symptoms are less severe and more varied, which could be more challenging for the model, explaining the higher error rates [52]. Figure 12 shows the misidentification by this model. Similarly to Figure 12a, Figure 12b represents the early onset of infection with additional difficulty in a highly close-up image. Figure 12c represents an error in the image with a busy background. We consider that a busy background in training data can improve generalisability in real-life conditions, as previously attempted [22,53]. Thus, there is still room for improvement in our ultra-lightweight model in this regard.

In addition, our systematic review of augmentation techniques suggests that generic combinations may introduce counterproductive noise for specialised agricultural tasks. Specifically, while standard multi-technique pipelines are often used as a default, our results indicate that they can simultaneously raise both accuracy and loss, signalling increased model uncertainty. We posit that for leaf pathology, geometric transformations, such as flipping and cropping, preserve the essential structural features of disease lesions, such as the distinctive brown angular patterns of leaf spot, whereas excessive photometric shifts (like extreme brightness or random noise) may obscure the subtle halo effects critical for identifying early-stage rust. This underscores the necessity of tailored augmentation strategies that prioritise the preservation of domain-specific visual markers over simple dataset expansion.

### 5.1. Implications for Agricultural Resource Management

The transition from centralised high-performance computing to decentralised Edge-AI carries significant implications for agricultural resource management. In traditional management models, the latency and cost of data transmission to the cloud often result in delayed interventions. By reducing the model footprint to 1.86 MB, a 70% reduction compared to standard architectures, this research facilitates the integration of sophisticated diagnostics into low-cost mobile management systems. This efficiency allows for precision agriculture practices where farmers can manage specific sections of a plot based on real-time data, optimising the application of fungicides and labour. Such data-driven decision-making not only reduces the operational overhead for smallholder farmers but also mitigates the environmental impact of broad-spectrum chemical use, aligning agricultural productivity with sustainable management goals.

### 5.2. Limitations

This work has a number of limitations, and caution is necessary not to overgeneralise the findings. The results are subject to the architectures and the ML settings described in this article for experimental convenience. Despite the remarkable performance of the models, the tuning and training process can be time-consuming. Although care was taken to avoid the models’ overfitting, the significantly small training data necessitates more diverse and robust sets to ensure that the proposed model adequately generalises across various datasets. Furthermore, the successful model construction of this work does not directly indicate real-time performance across crop types since the training, validation, and testing are based solely on the common bean plant cultivated in Uganda. Other crop types or bean plant varieties may exhibit different results.

## 6. Conclusions

Automated image-based disease classification aims to reduce yield loss and ensure global food security. We investigated the feasibility of a hybrid CNN-LSTM architecture for this task. In our two-phase experiments, we introduced LSTM-based ultra-lightweight custom models and pre-trained EfficientNet. Our primary results show that Bean-CNN-LSTM consistently outperforms Bean-CNN with 2.56% to 5.64% higher best test accuracy, depending on the training sets. The best Bean-CNN-LSTM model of 1.86 MB in size achieved an accuracy of 94.36%. This result inspired us to conduct further experiments with a pre-trained image recognition model, EfficientNet. In these experiments, we discovered that our modified EfficientNet models, trained on significantly larger data, improved the performance further. Both an FC-based and an LSTM-based model achieved 99.22% accuracy and F1 score. Although there are no significant gains in training efficiency, integrating an LSTM with CNN contributed to streamlining the model and enhancing performance. Nevertheless, we found that complex backgrounds and disease pattern variations are challenging, especially for our lightweight models. For further improvement, advanced feature extraction methods using specialised filters or segmentation are worth exploring. Also, comparing other RNN families, such as a gated recurrent unit (GRU), could shed light on discovering more efficient models. While the lightweight architecture demonstrates strong theoretical potential for edge deployment, physical hardware testing was beyond the scope of this study. Consequently, empirical evaluations of inference latency, peak RAM utilisation, and energy consumption on physical edge devices remain important targets for future work. 

## Figures and Tables

**Figure 1:**
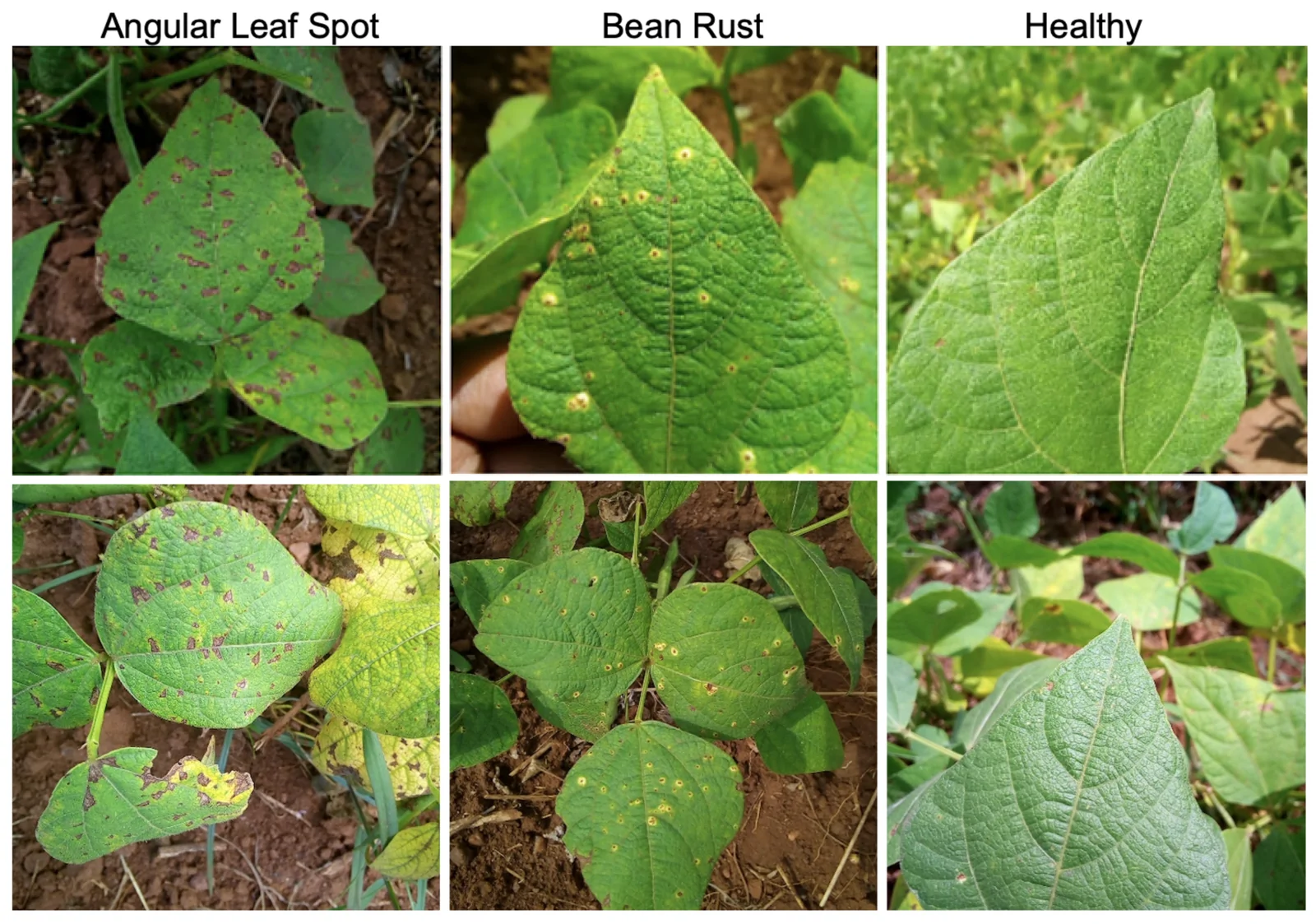
Three classes of bean leaf images in the *ibean* dataset.

**Figure 2:**
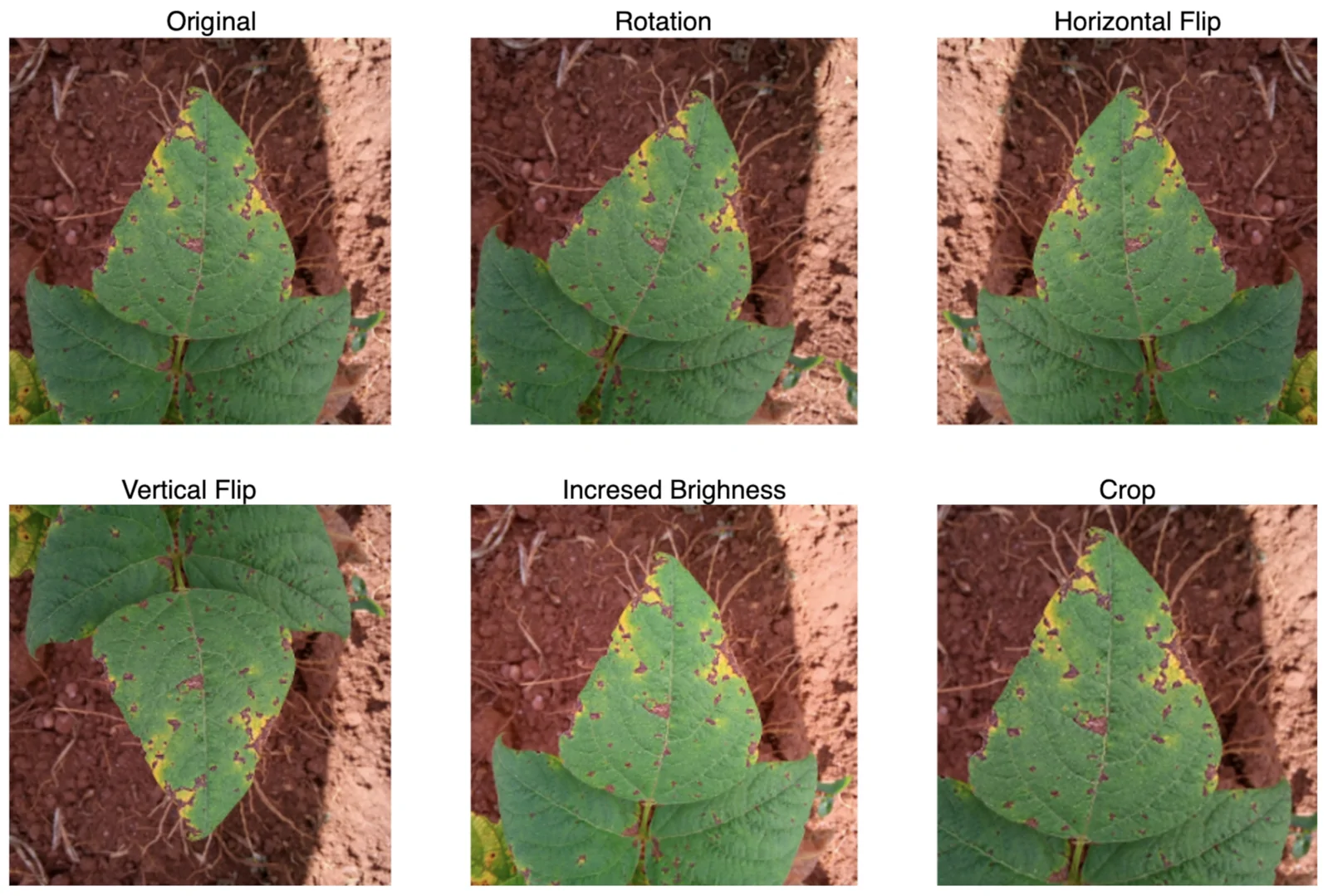
Augmentation effect.

**Figure 3:**
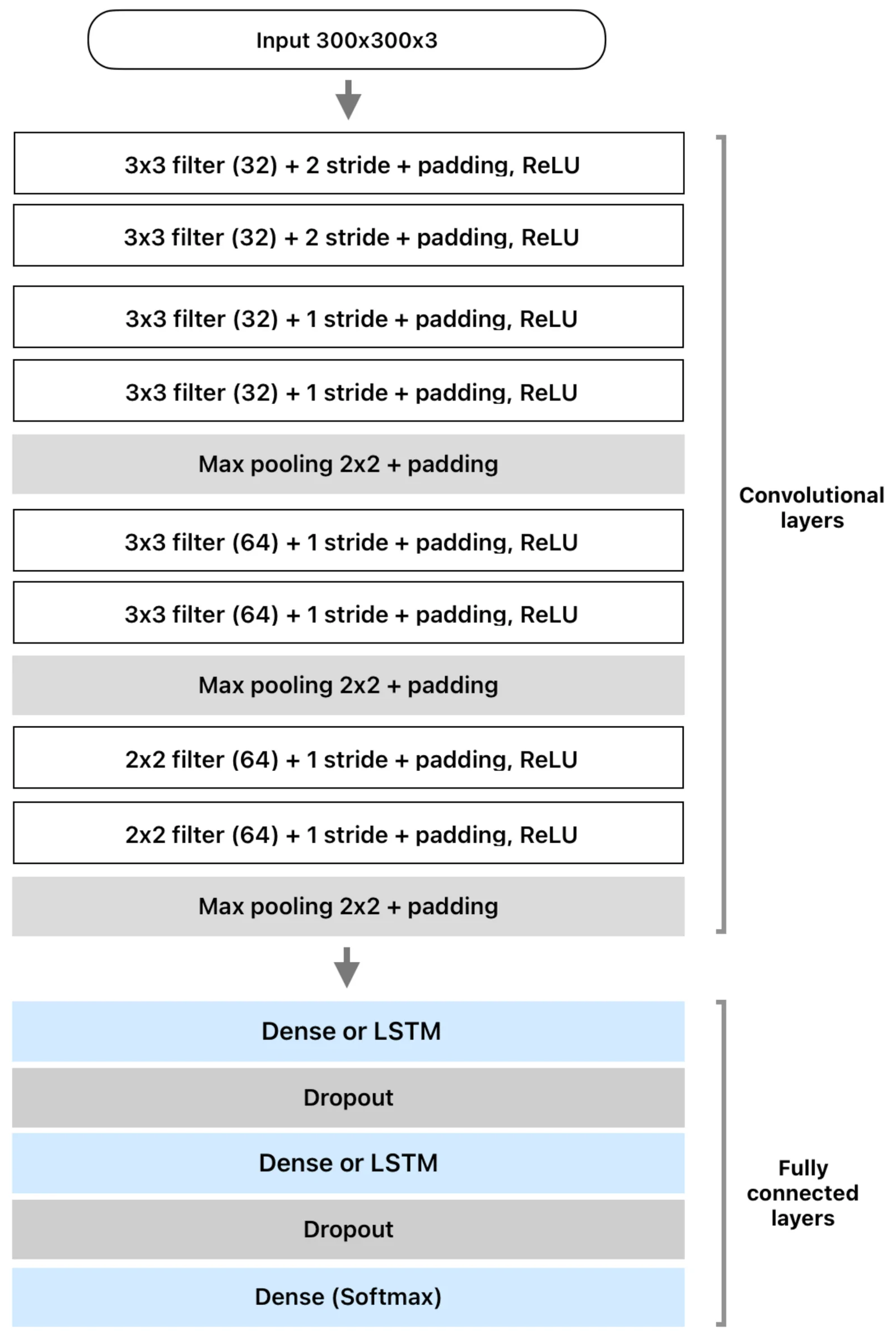
Custom architecture of lightweight models.

**Figure 4:**
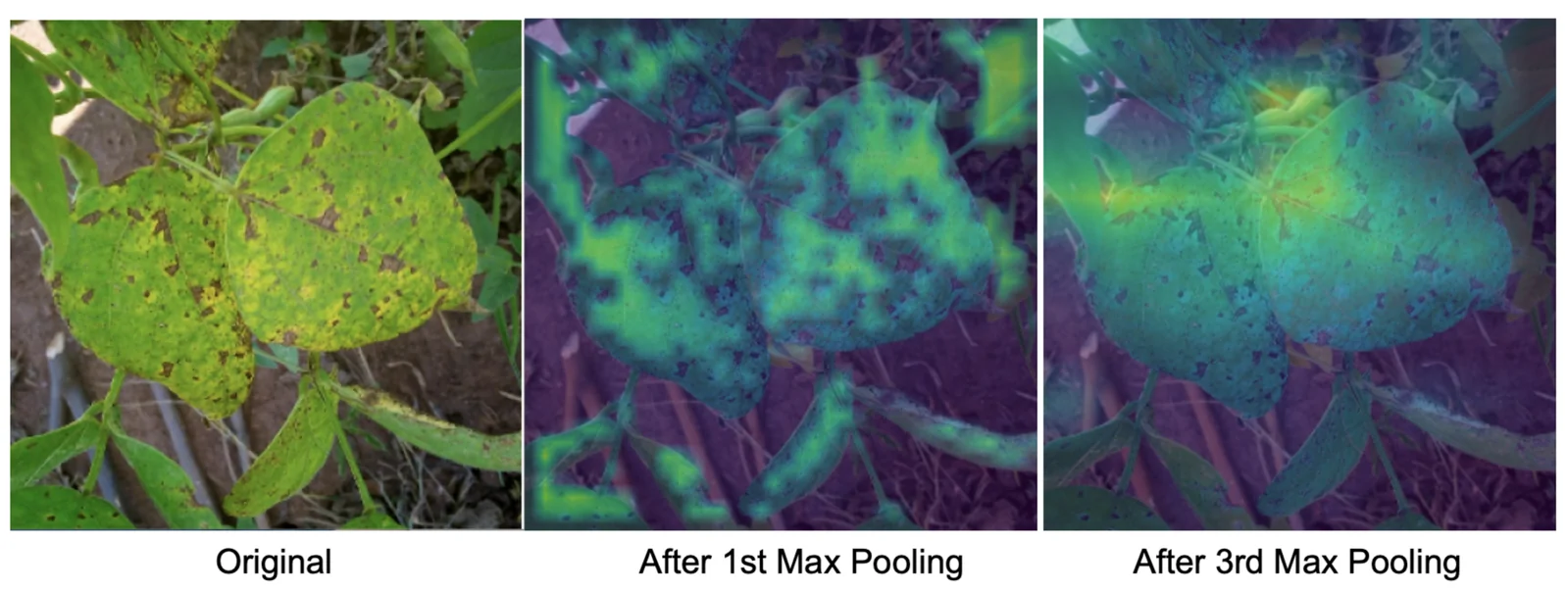
Activation map by a custom lightweight model.

**Figure 5:**
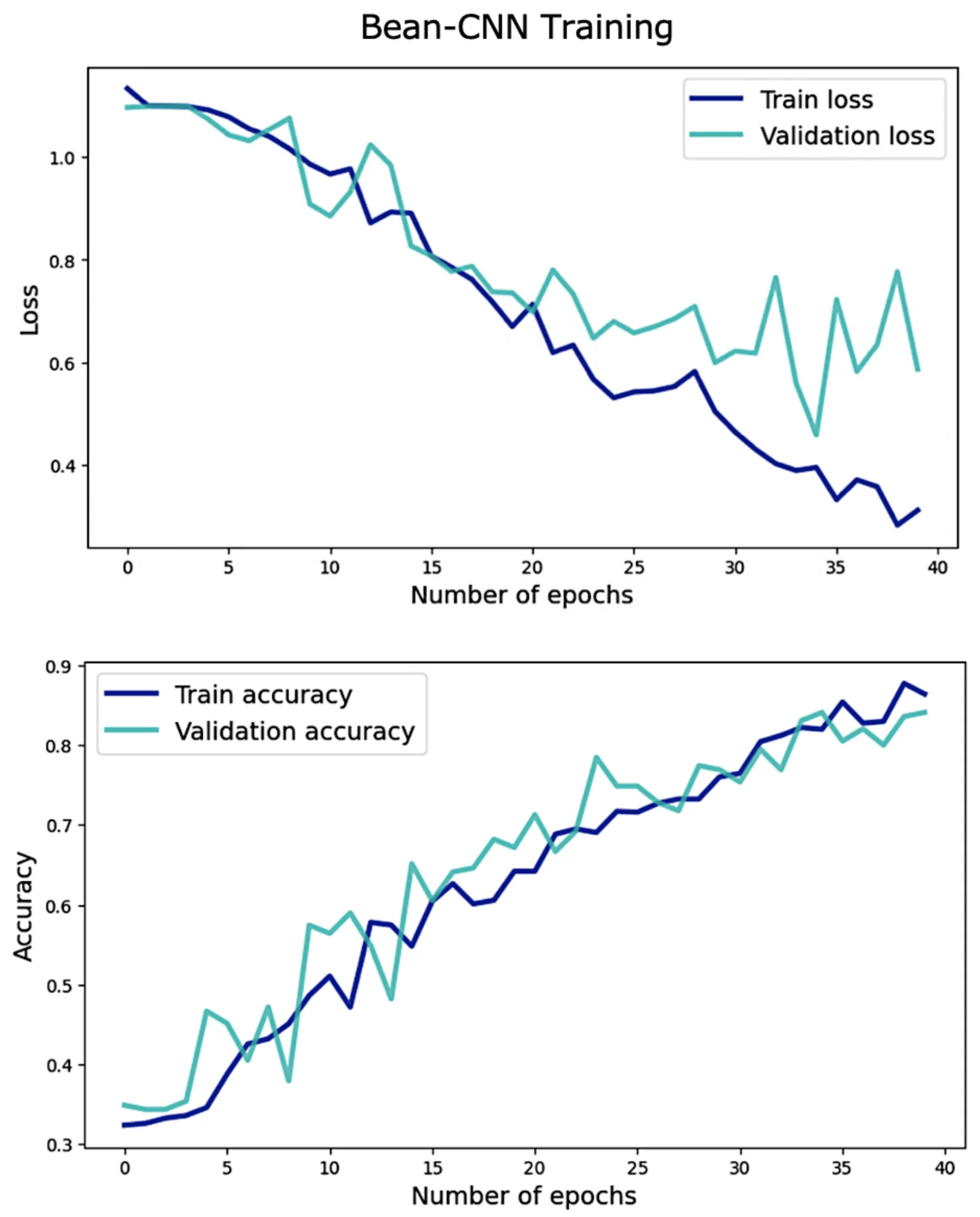
Training Bean-CNN on the unaugmented set.

**Figure 6:**
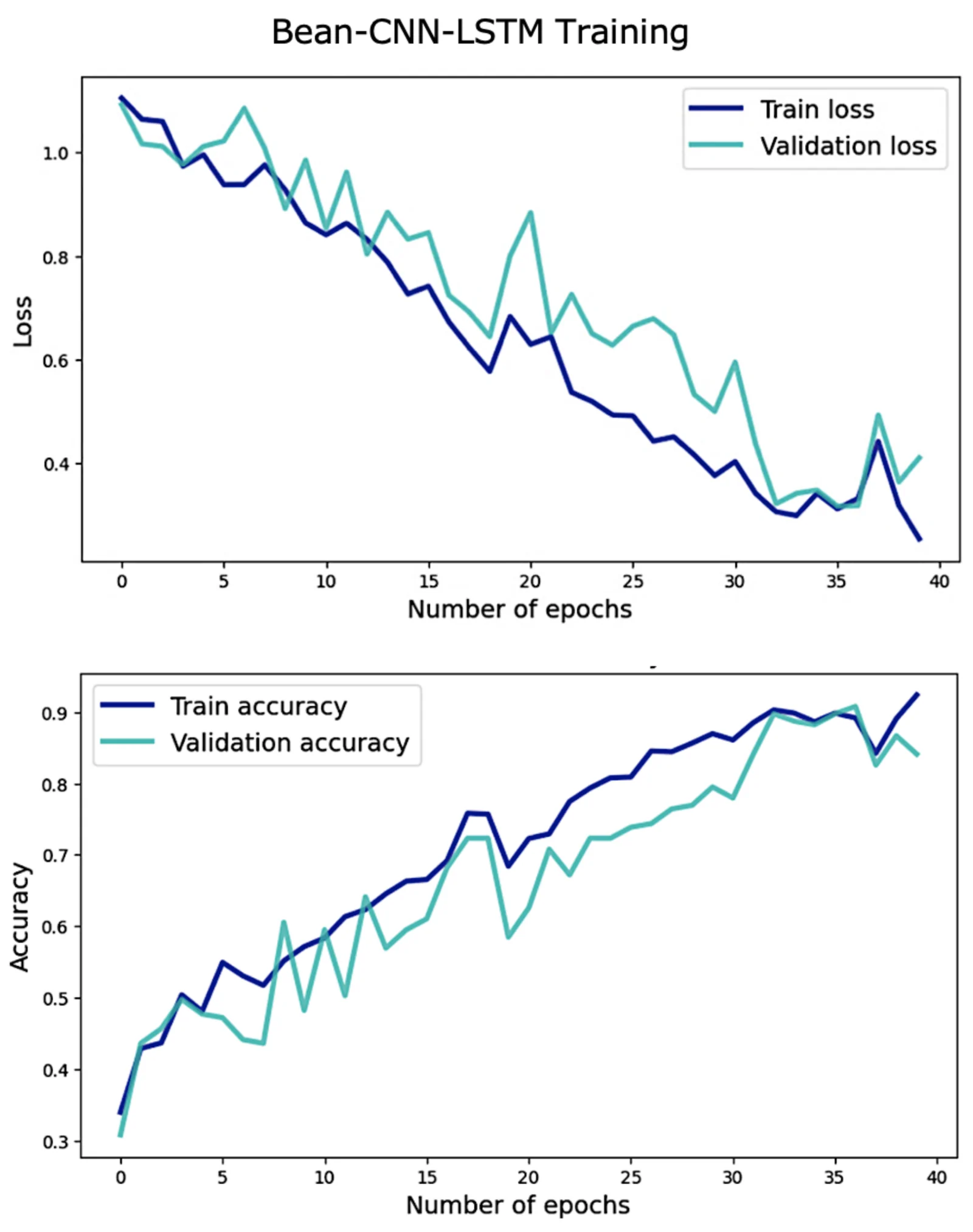
Training Bean-CNN-LSTM on the unaugmented set.

**Figure 7:**
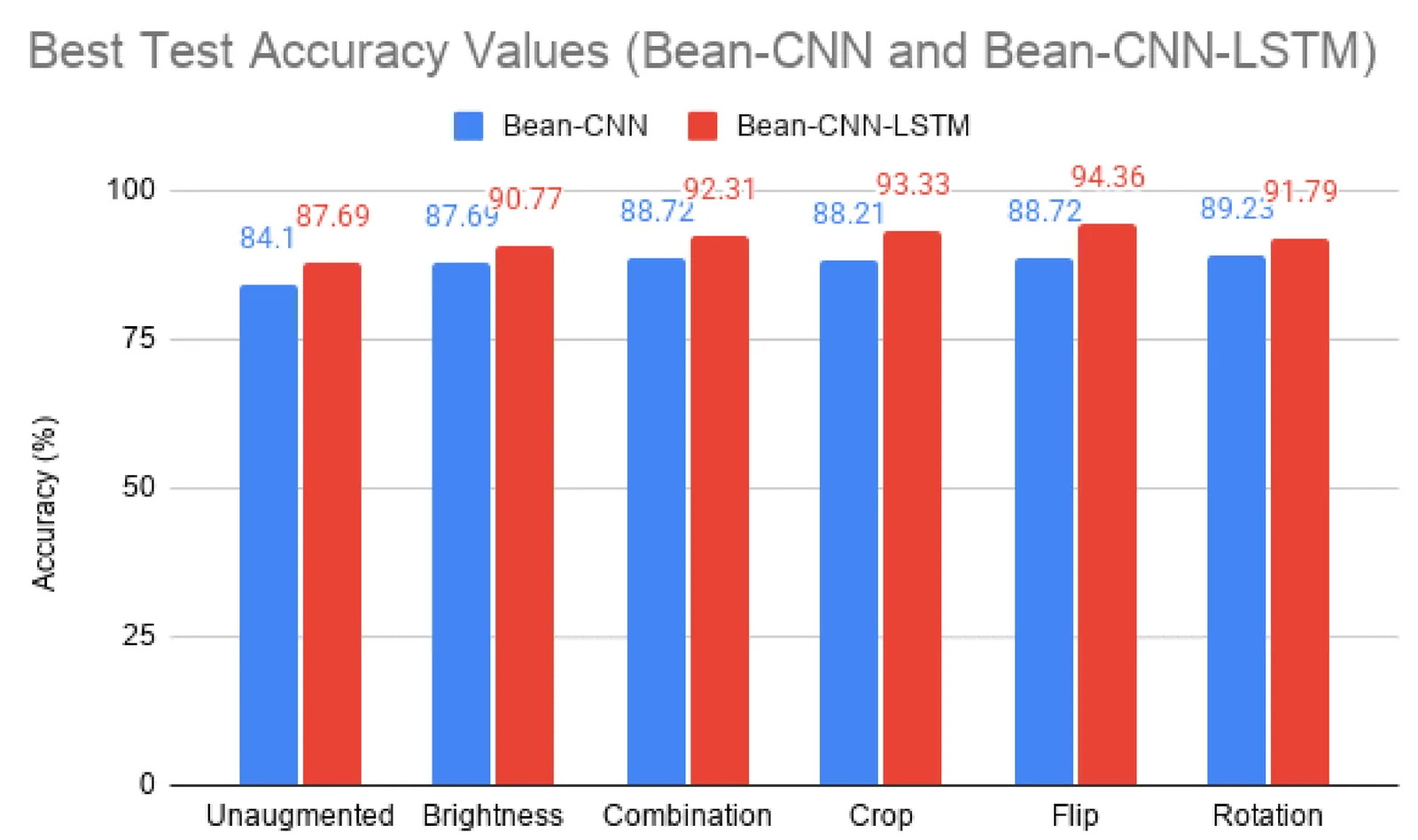
Graph representation of the best test accuracy: Comparison of two custom lightweight architectures.

**Figure 8:**
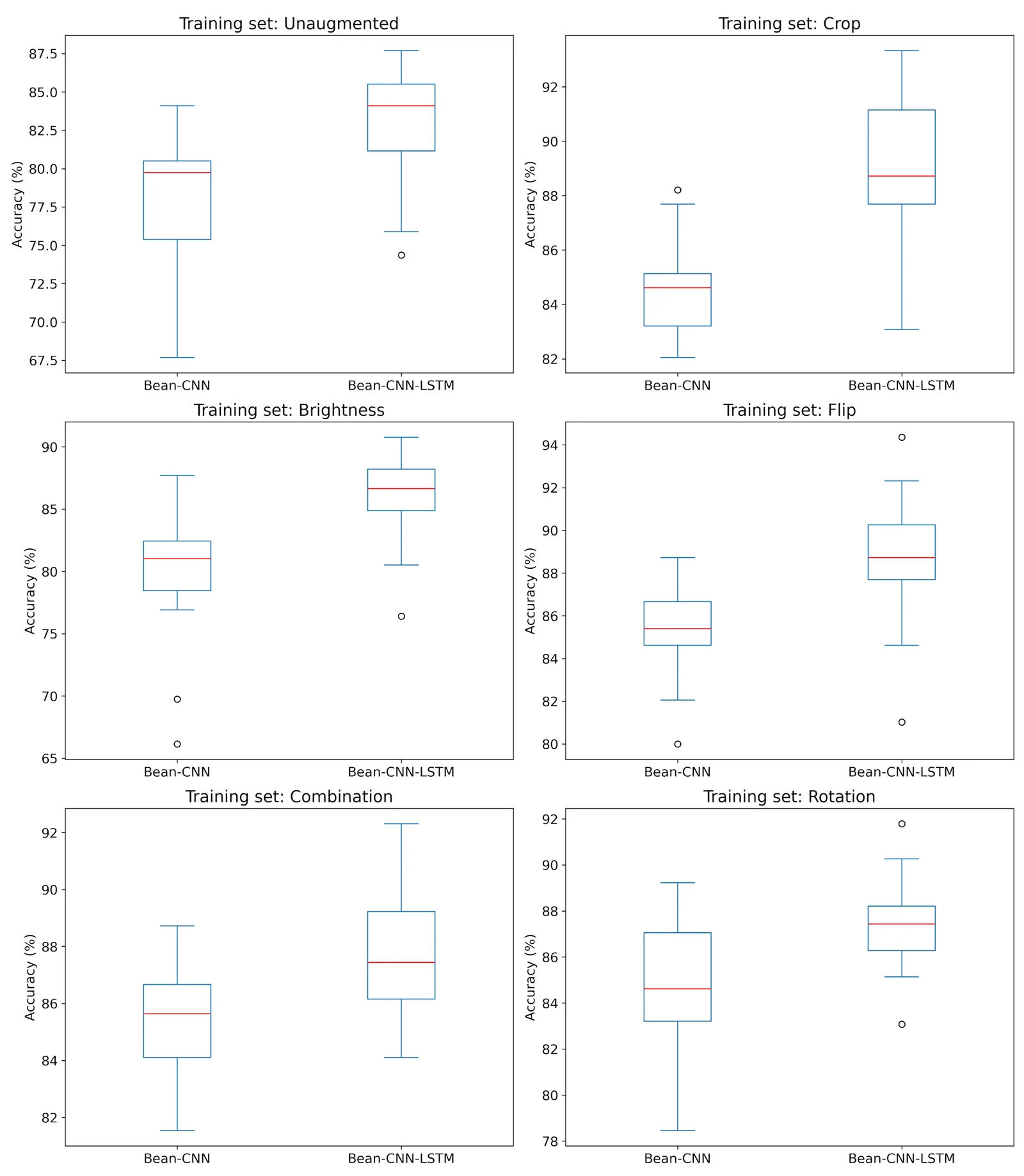
Box plots showing the average test accuracy on each training set: The red line indicates the median value, and a box represents the distribution between 25 and 75 percentiles. Small hollow circles indicate outliers.

**Figure 9:**
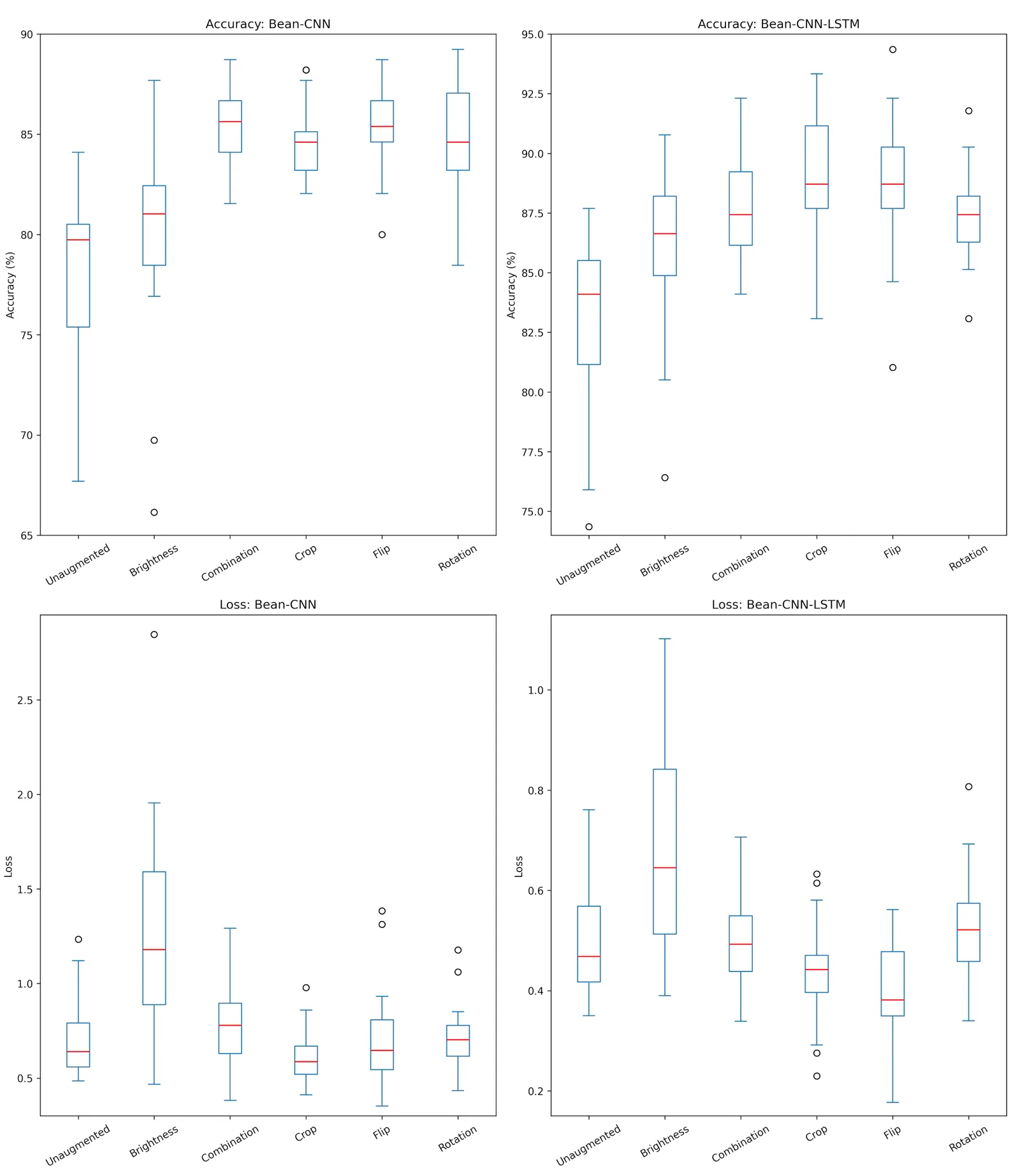
Box-plot representation: The average performance of our lightweight custom models (Bean-CNN and Bean-CNN-LSTM) from 30 training runs.

**Figure 10:**
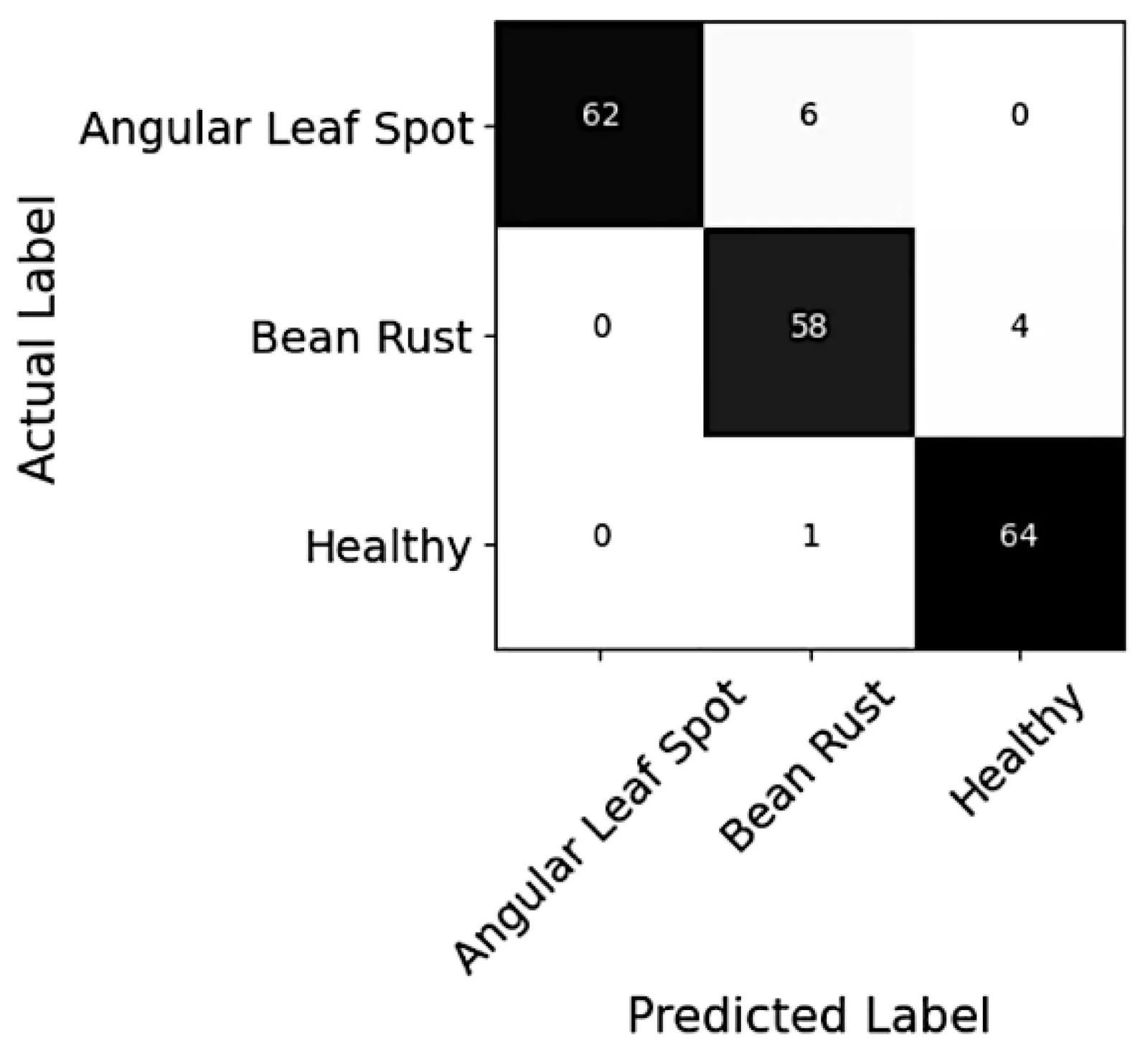
Confusion matrix: The best Bean-CNN-LSTM model.

**Figure 11:**
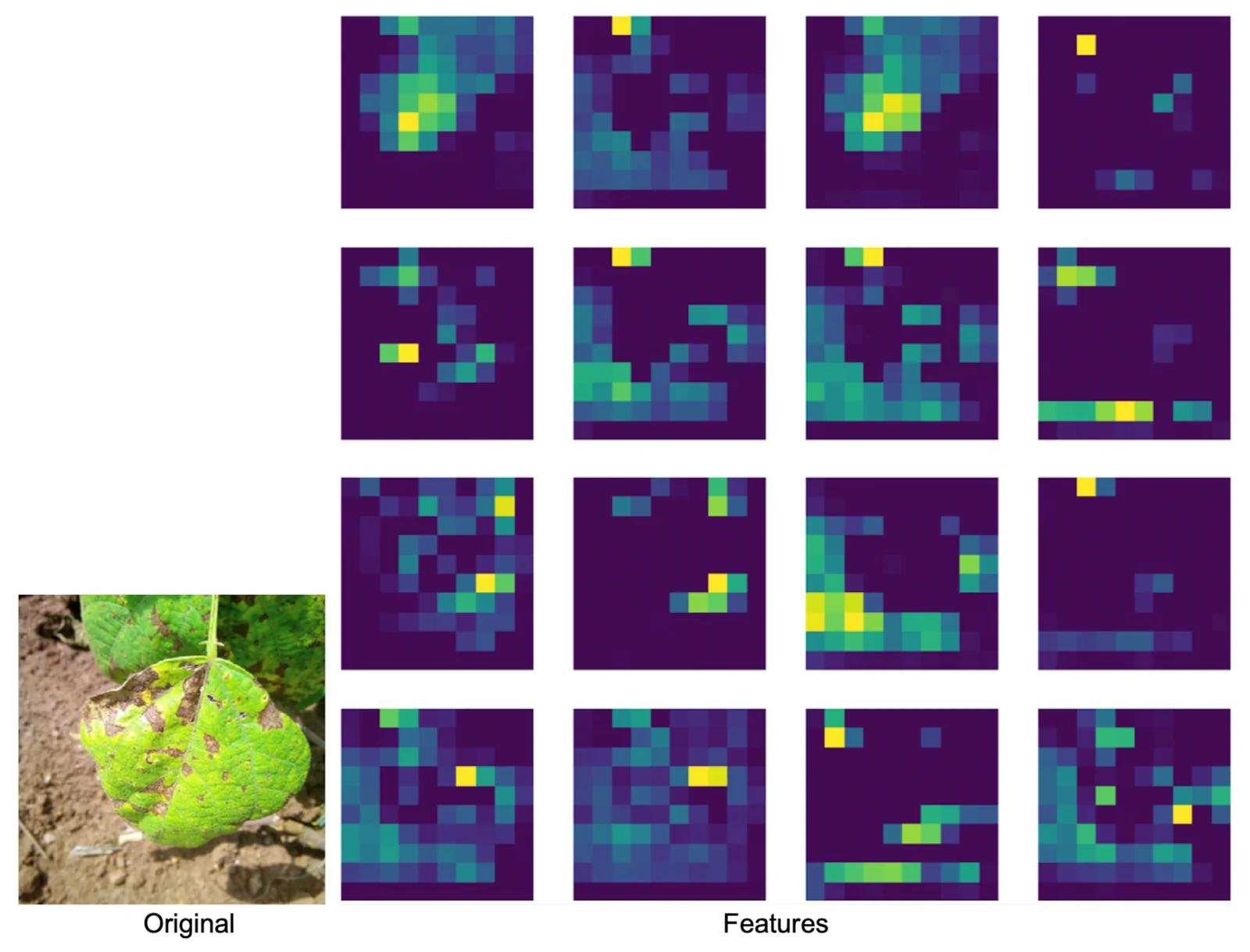
GradCAM feature maps. The heatmaps indicate that the model correctly prioritises the necrotic centres of the lesions (yellow patches indicate high activation) rather than the leaf edges or image background (dark-blue regions), validating the management reliability of the system.

**Figure 12:**
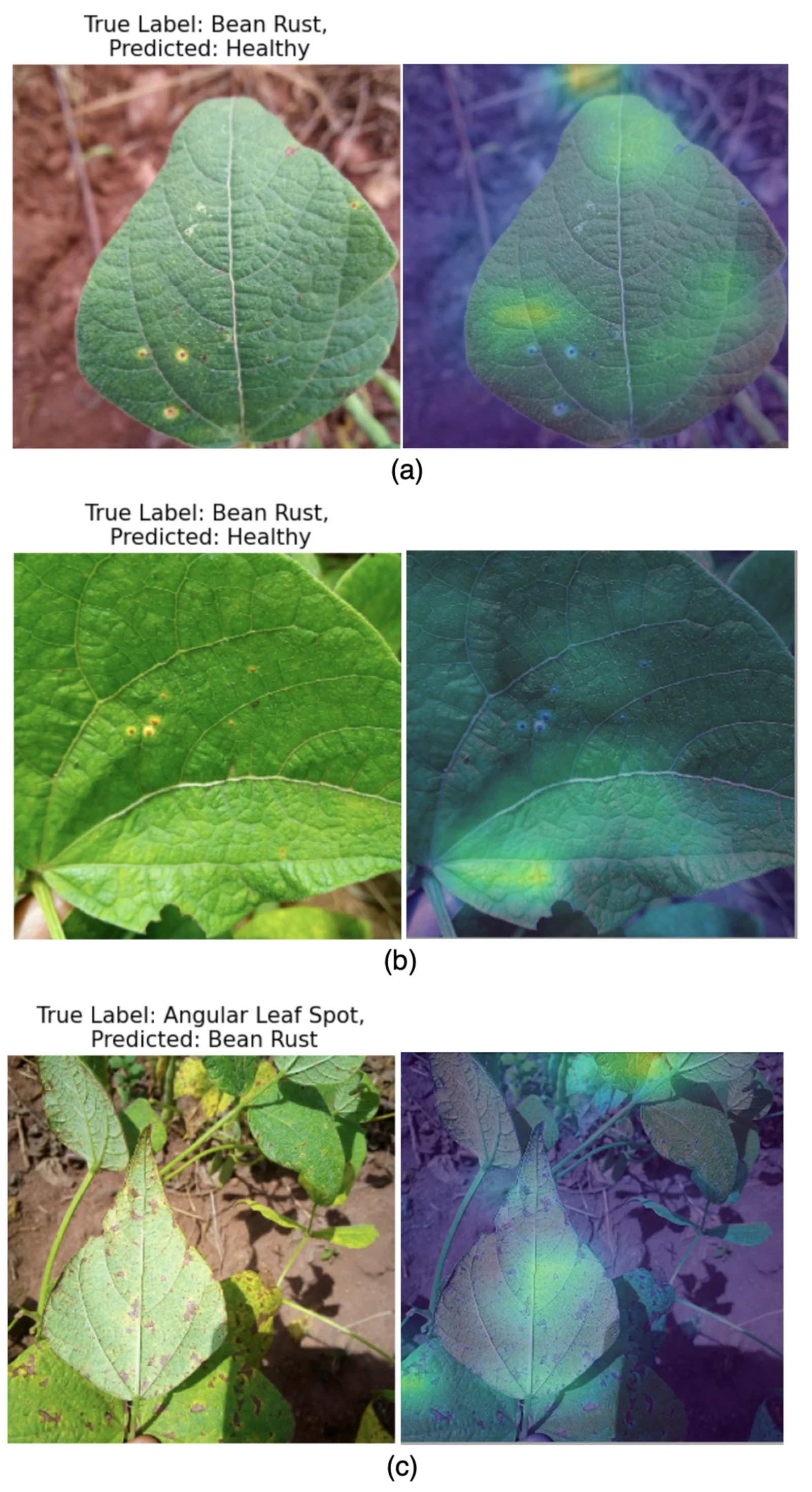
Misclassified images. The first column indicates the raw images, while the second column shows the predicted image activation. (**a**) True label: Bean rust; Predicted label: Healthy. (**b**) True label: Bean rust; Predicted label: Healthy. (**c**) True label: Angular leaf spot; Predicted label: Bean rust.

**Table 2:** Configuration of custom lightweight models.

Parameter	Bean-CNN	Bean-CNN-LSTM
Learning rate	1 $\times \;10^{-3}$	1 $\times \;10^{-3}$
Epochs	40	40
Optimiser	Adam	Adam
Batch size	32	32
Final Conv + Pooling dim.	10×10×64	10×10×64
Reshape dim.	6400 (flattened)	100×64
Traversal order	–	Row-major (scan-line)
Sequential layer (1st and 2nd)	64 units and 8 units	64 units and 16 units
Dropout rate	0.3	0.3

**Table 3:** Top-1 Test performance of custom lightweight models: Results across training sets—the best shown in bold.

Bean-CNN				
Training Set	Accuracy	Loss	F1 Score	MCC
Unaugmented	0.8410	0.5180	0.8410	0.7614
Brightness	0.8769	0.5180	0.8751	0.8163
Combination	0.8872	0.6561	0.8882	0.8329
Crop	0.8821	0.4597	0.8844	0.8267
Flip	0.8872	0.5089	0.8867	0.8307
Rotation	**0.8923**	0.5130	**0.8913**	**0.8391**
**Bean-CNN-LSTM**				
Training set	Accuracy	Loss	F1 Score	MCC
Unaugmented	0.8769	0.3773	0.8737	0.8173
Brightness	0.9077	0.3902	0.9074	0.8615
Combination	0.9231	0.4116	0.9234	0.8856
Crop	0.9333	0.2757	0.9332	0.9000
Flip	**0.9436**	0.1769	**0.9438**	**0.9164**
Rotation	0.9179	0.3398	0.9168	0.8776

**Table 4:** Mean and standard deviation (Std.) values across the 30 experiment runs for Bean-CNN and Bean-CNN-LSTM.

Bean-CNN						
	Accuracy		F1 Score		MCC	
Training Set	Mean	Std.	Mean	Std.	Mean	Std.
Unaugmented	0.7808	0.0403	0.7778	0.0443	0.6776	0.0562
Brightness	0.8026	0.0444	0.8021	0.0446	0.7092	0.0622
Combination	0.8525	0.0174	0.8518	0.0177	0.7855	0.0214
Crop	0.8460	0.0170	0.8492	0.0174	0.7732	0.0246
Flip	0.8540	0.0214	0.8533	0.0220	0.7849	0.0314
Rotation	0.8475	0.0266	0.8470	0.0276	0.7740	0.0370
**Bean-CNN-LSTM**						
	Accuracy		F1 Score		MCC	
Training set	Mean	Std.	Mean	Std.	Mean	Std.
Unaugmented	0.8275	0.0383	0.8274	0.0381	0.7470	0.0545
Brightness	0.8624	0.0308	0.8636	0.0299	0.7968	0.0436
Combination	0.8770	0.0205	0.8760	0.0211	0.8165	0.0341
Crop	0.8875	0.0271	0.8744	0.0802	0.8331	0.0401
Flip	0.8865	0.0250	0.8854	0.0263	0.8322	0.0362
Rotation	0.8744	0.0183	0.8740	0.0183	0.8136	0.0267

**Table 5:** Paired 2-tailed *t*-test of the Accuracy, F1 score and MCC values for Bean-CNN versus Bean-CNN-LSTM across the 30 experiments. N=30 degree of freedom =29.

Training Set	Accuracy	F1 Score	MCC
Unaugmented	$3.52\times 10^{-6}$	$2.49\times 10^{-6}$	$2.55\times 10^{-6}$
Brightness	$1.29\times 10^{-5}$	$7.81\times 10^{-6}$	$7.53\times 10^{-6}$
Combination	$1.25\times 10^{-5}$	$2.60\times 10^{-5}$	$3.02\times 10^{-4}$
Crop	$1.33\times 10^{-7}$	$9.96\times 10^{-2}$	$2.11\times 10^{-7}$
Flip	$8.13\times 10^{-6}$	$1.59\times 10^{-5}$	$1.18\times 10^{-5}$
Rotation	$1.19\times 10^{-4}$	$1.25\times 10^{-4}$	$7.14\times 10^{-5}$

**Table 6:** Performance of the best Bean-CNN-LSTM model.

Class	Precision (%)	Recall (%)	F1 Score (%)
Angular leaf spot	100.00	91.18	95.38
Bean rust	89.23	93.55	91.34
Healthy	94.12	98.46	96.24
Overall Acc. (%)	94.36		
Overall F1 (%)	94.38		

**Table 7:** Classification scores of both the EfficientNetB7+FC and EfficientNetB7+LSTM Models.

Class	Precision (%)	Recall (%)	F1 Score (%)
Angular leaf spot	97.73	100.00	98.85
Bean rust	100.00	97.67	98.82
Healthy	100.00	100.00	100.00
Overall Acc. (%)	99.22		
Overall F1 (%)	99.22		

**Table 8:** Comparison with previous works.

Source	Method	Acc.; F1 Score (%)	Train/Test Size	Augmentation
Abed et al., 2021 [46]	DenseNet121	91.02; –	9324/259	flip and rotation
Elfatimi et al., 2022 [9]	MobileNetV2	92.97; 92.94	1034/128	none
Singh et al., 2023 [47]	EfficientnetB6	91.74; –	1034/128	none
Önler 2023 [8]	MobileNetV2	99.24; –	1034/128	blur, brightness, crop, flip, and rotation
Sunyoto et al., 2024 [48]	DenseNet121	96.90; 97.00	1034/128	rotation and zoom
Jain & Aneja 2025 [49]	InceptionV3	91.00; 91.00	693/149	brightness, flip, rotation, and zoom
Karthik et al., 2025 [50]	Transformer+CNN	97.66; 97.67	13,442/128	flip, rotation, and translation
**Ours**	**Bean-CNN-LSTM**	**94.36**; **94.38**	41,360/128	blur, brightness, flip, rotation, and scaling
	**EfficientNetB7+FC**	**99.22**; **99.22**	as above	as above
	**EfficientNetB7+LSTM**	**99.22**; **99.22**	as above	as above

**Table 9:** Traversal strategies comparison for Bean-CNN-LSTM.

Traversal Strategy	Tensor Shape	Test Accuracy (%)	Test F1 (%)	Test MCC (%)
Row-major (scan-line) [proposed]	100×64	89.74	89.64	85.11
Column-major	100×64	88.72	88.70	83.32
Patch-wise (2×2)	25×256	86.67	86.58	80.15

**Table 10:** Parameter-matched architectural check.

Architecture	Classification Head	Param. Count	Model Size (MB)	Test Accuracy (%)	Test F1 (%)	Test MCC (%)
Bean-CNN (Baseline)	Standard FC	527,171	6.11	84.62	84.48	77.24
Bean-CNN-Compact (Control)	Bottleneck FC	155,219	1.86	82.56	82.48	74.12
Bean-CNN-LSTM (Proposed)	Spatial-to-Seq LSTM	155,219	1.86	89.74	89.64	85.11

**Table 12:** Summary of models.

Models	Model Size (MB)	Total Params	Trainable Params	Best Accuracy/F1 (%)
Bean-CNN	6.11	527,171	527,171	89.23/89.13
Bean-CNN-LSTM	1.86	155,219	155,219	94.36/94.38
EfficientNetB7+FC	260.56	67,825,595	4,039,035	99.22/99.22
EfficientNetB7+LSTM	250.20	65,165,139	1,377,667/52,844,795	99.22/99.22

## Data Availability

The originally downloaded (OD) *ibean* dataset is available from [27] while the augmented dataset curated in this study have been made openly available on IEEE Dataport https://dx.doi.org/10.21227/4k7y-vs03 and on Kaggle https://www.kaggle.com/datasets/akinyemijoseph/ibean-aug.

## References

[1] Pamela P., Mawejje D., Ugen M. (2014). Severity of angular leaf spot and rust diseases on common beans in Central Uganda. Uganda J. Agric. Sci..

[2] Venbrux M., Crauwels S., Rediers H. (2023). Current and emerging trends in techniques for plant pathogen detection. Front. Plant Sci..

[3] Mahlein A.K. (2016). Plant disease detection by imaging sensors–parallels and specific demands for precision agriculture and plant phenotyping. Plant Dis..

[4] Rahunathan L., Sivabalaselvamani D., Elakkiya E., Madhumitha M., Kumaresh K. Recognition of Bean Leaf Diseases Using Neural Network and Machine Learning Techniques. Proceedings of the 2023 3rd International Conference on Smart Data Intelligence (ICSMDI).

[5] Slimani H. (2023). Artificial Intelligence-based Detection of Fava Bean Rust Disease in Agricultural Settings: An Innovative Approach. Int. J. Adv. Comput. Sci. Appl..

[6] Islam Z., Islam M., Amanullah A. (2020). A combined deep CNN-LSTM network for the detection of novel coronavirus (COVID-19) using X-ray images. Inform. Med. Unlocked.

[7] Donahue J., Hendricks L.A., Rohrbach M., Venugopalan S., Guadarrama S., Saenko K., Darrell T. (2017). Long-Term Recurrent Convolutional Networks for Visual Recognition and Description. IEEE Trans. Pattern Anal. Mach. Intell..

[8] Önler E. (2023). Feature fusion based artificial neural network model for disease detection of bean leaves. Electron. Res. Arch..

[9] Elfatimi E., Eryigit R., Elfatimi L. (2022). Beans Leaf Diseases Classification Using MobileNet Models. IEEE Access.

[10] Tan M., Le Q. Efficientnet: Rethinking model scaling for convolutional neural networks. Proceedings of the International Conference on Machine Learning.

[11] Jian Z., Wei Z. Support vector machine for recognition of cucumber leaf diseases. Proceedings of the 2010 2nd International Conference on Advanced Computer Control.

[12] Lu Y., Yi S., Zeng N., Liu Y., Zhang Y. (2017). Identification of rice diseases using deep convolutional neural networks. Neurocomputing.

[13] Geetharamani G., Arun Pandian J. (2019). Identification of plant leaf diseases using a nine-layer deep convolutional neural network. Comput. Electr. Eng..

[14] Mohanty S.P. (2016). Using Deep Learning for Image-Based Plant Disease Detection. Front. Plant Sci..

[15] Patil M.A., Manohar M. (2023). Plant Leaf Disease Classification Using Optimal Tuned Hybrid LSTM-CNN Model. SN Comput. Sci..

[16] Devi E., Gopi S., Padmavathi U., Arumugam S.R., Premnath S., Muralitharan D. Plant Disease Classification using CNN-LSTM Techniques. Proceedings of the 2023 5th International Conference on Smart Systems and Inventive Technology (ICSSIT).

[17] Haque M.A., Deb C.K., Gole P., Karmakar S., Dheeraj A., Din Shah M.U., Dutta S., Kumar M.K.P., Marwaha S. (2025). An enhanced vision transformer network for efficient and accurate crop disease detection. Expert Syst. Appl..

[18] Abade A., Ferreira P.A., de Barros Vidal F. (2021). Plant diseases recognition on images using convolutional neural networks: A systematic review. Comput. Electron. Agric..

[19] Singla S., Gupta R. Deep Learning based Bean Leaf Lesion Classification utilizing EfficientNetV2-S. Proceedings of the 2024 8th International Conference on I-SMAC (IoT in Social, Mobile, Analytics and Cloud) (I-SMAC).

[20] Rodríguez-Lira D.C., Córdova-Esparza D.M., Álvarez Alvarado J.M., Romero-González J.A., Terven J., Rodríguez-Reséndiz J. (2024). Comparative Analysis of YOLO Models for Bean Leaf Disease Detection in Natural Environments. AgriEngineering.

[21] Hohman F., Kery M.B., Ren D., Moritz D. Model Compression in Practice: Lessons Learned from Practitioners Creating On-device Machine Learning Experiences. Proceedings of the CHI ’24: CHI Conference on Human Factors in Computing Systems.

[22] Sun H., Xu H., Liu B., He D., He J., Zhang H., Geng N. (2021). MEAN-SSD: A novel real-time detector for apple leaf diseases using improved light-weight convolutional neural networks. Comput. Electron. Agric..

[23] Arsenovic M., Karanovic M., Sladojevic S., Anderla A., Stefanovic D. (2019). Solving Current Limitations of Deep Learning Based Approaches for Plant Disease Detection. Symmetry.

[24] Yang S., Xiao W., Zhang M., Guo S., Zhao J., Shen F. (2023). Image Data Augmentation for Deep Learning: A Survey. arXiv.

[25] Taylor L., Nitschke G. Improving Deep Learning with Generic Data Augmentation. Proceedings of the 2018 IEEE Symposium Series on Computational Intelligence (SSCI).

[26] Shorten C., Khoshgoftaar T.M. (2019). A survey on Image Data Augmentation for Deep Learning. J. Big Data.

[27] (2020). AI-Lab-Makerere/ibean. https://github.com/AI-Lab-Makerere/ibean/.

[28] Muimba-Kankolongo A. (2018). Food Crop Production by Smallholder Farmers in Southern Africa.

[29] Deng L., Platt J.C. (2014). Ensemble deep learnig for speech recognition. Interspeech.

[30] Sainath T.N., Vinyals O., Senior A., Sak H. Convolutional, Long Short-Term Memory, fully connected Deep Neural Networks. Proceedings of the 2015 IEEE International Conference on Acoustics, Speech and Signal Processing (ICASSP).

[31] Ercolano G., Rossi S. (2021). Combining CNN and LSTM for activity of daily living recognition with a 3D matrix skeleton representation. Intell. Serv. Robot..

[32] Visin F., Kastner K., Cho K., Matteucci M., Courville A., Bengio Y. (2015). ReNet: A Recurrent Neural Network Based Alternative to Convolutional Networks. arXiv.

[33] Van Den Oord A., Kalchbrenner N., Kavukcuoglu K. Pixel recurrent neural networks. Proceedings of the ICML’16: 33rd International Conference on International Conference on Machine Learning—Volume 48.

[34] Dosovitskiy A., Beyer L., Kolesnikov A., Weissenborn D., Zhai X., Unterthiner T., Dehghani M., Minderer M., Heigold G., Gelly S. (2021). An Image is Worth 16 × 16 Words: Transformers for Image Recognition at Scale. arXiv.

[35] Simonyan K., Zisserman A. Very Deep Convolutional Networks for Large-Scale Image Recognition. Proceedings of the 3rd International Conference on Learning Representations, ICLR 2015.

[36] Zhang X., Han N., Zhang J. (2024). Comparative analysis of VGG, ResNet, and GoogLeNet architectures evaluating performance, computational efficiency, and convergence rates. Appl. Comput. Eng..

[37] Nixon M.S., Aguado A.A. (2020). Feature Extraction and Image Processing for Computer Vision.

[38] He K., Zhang X., Ren S., Sun J. Delving Deep into Rectifiers: Surpassing Human-Level Performance on ImageNet Classification. Proceedings of the 2015 IEEE International Conference on Computer Vision (ICCV).

[39] Hochreiter S., Schmidhuber J. (1997). Long Short-Term Memory. Neural Comput..

[40] Gers F.A., Schmidhuber J., Cummins F. (2000). Learning to Forget: Continual Prediction with LSTM. Neural Comput..

[41] Tharwat A. (2021). Classification assessment methods. Appl. Comput. Inform..

[42] Chicco D., Jurman G. (2020). The advantages of the Matthews correlation coefficient (MCC) over F1 score and accuracy in binary classification evaluation. BMC Genom..

[43] Selvaraju R.R., Cogswell M., Das A., Vedantam R., Parikh D., Batra D. Grad-CAM: Visual Explanations from Deep Networks via Gradient-Based Localization. Proceedings of the 2017 IEEE International Conference on Computer Vision (ICCV).

[44] Goodfellow I., Bengio Y., Courville A. (2016). Deep Learning.

[45] Kingma D.P., Ba J. (2014). Adam: A method for stochastic optimization. arXiv.

[46] Abed S.H., Al-Waisy A.S., Mohammed H.J., Al-Fahdawi S. (2021). A modern deep learning framework in robot vision for automated bean leaves diseases detection. Int. J. Intell. Robot. Appl..

[47] Singh V., Chug A., Singh A.P. (2023). Classification of beans leaf diseases using fine tuned cnn model. Procedia Comput. Sci..

[48] Sunyoto A., Ariatmanto D., Noviyanto Innovative Solutions for Bean Leaf Disease Detection Using Deep Learning. Proceedings of the 2024 IEEE International Conference on Artificial Intelligence and Mechatronics Systems (AIMS).

[49] Jain E., Aneja A. Automated Detection and Classification of Bean Leaf Diseases using InceptionV3: A Deep Learning Approach. Proceedings of the 2025 International Conference on Electronics and Renewable Systems (ICEARS).

[50] Karthik R., Aswin R., Geetha K.S., Suganthi K. (2025). An Explainable Deep Learning Network With Transformer and Custom CNN for Bean Leaf Disease Classification. IEEE Access.

[51] Kahatapitiya K., Rodrigo R. Exploiting the Redundancy in Convolutional Filters for Parameter Reduction. Proceedings of the 2021 IEEE Winter Conference on Applications of Computer Vision (WACV).

[52] Barbedo J.G.A. (2016). A review on the main challenges in automatic plant disease identification based on visible range images. Biosyst. Eng..

[53] Fenu G., Malloci F.M. (2021). DiaMOS Plant: A Dataset for Diagnosis and Monitoring Plant Disease. Agronomy.

